# Enhanced Nonlinear Optical Properties and Optical Limiting Performance of Perylenediimide Derivative/Semiconductor Nanocomposites Under Femtosecond Laser Light Excitation

**DOI:** 10.3390/ma19122587

**Published:** 2026-06-16

**Authors:** Tarek Mohamed, Majed H. El-Motlak, Fatma Abdel Samad, Mohamed E. El-Khouly, Sulaiman Wadi Harun, Alaa Mahmoud

**Affiliations:** 1 Laser Institute for Research and Applications LIRA, Beni-Suef University, Beni-Suef 62511, Egypt; 2 Department of Engineering, Faculty of Advanced Technology and Multidiscipline, Universitas Airlangga, Surabaya 60115, Indonesia; 3 Al Anbar Health Directorate, Training and Human Development Centre, Ramadi 31001, Iraq; 4 Faculty of Basic and Applied Sciences, Egypt-Japan University of Science and Technology, New Borg El-Arab City 21934, Egypt; 5 Department of Electrical Engineering, Faculty of Engineering, University of Malaya, Kuala Lumpur 50603, Malaysia

**Keywords:** NLO properties, perylenediimide, titanium dioxide, zinc oxide, femtosecond laser, Z-scan technique, laser ablation, optical limiting

## Abstract

The linear and third-order nonlinear optical (NLO) properties of a water-soluble perylenediimide derivative, N,N′-di(2-(trimethylammonium iodide) ethylene) perylenediimide (TAIPDI), doped with semiconductor nanoparticles (NPs), were systematically investigated under femtosecond laser excitation. ZnO and TiO_2_ NPs were synthesized using a pulsed laser ablation technique. Nanocomposite systems were prepared by incorporating different concentrations of ZnO and TiO_2_ NPs into the TAIPDI dye solution. The optical properties were characterized using UV–visible absorption spectroscopy together with open- and closed-aperture Z-scan measurements at 800 nm. Linear absorption measurements revealed concentration-dependent modifications in the optical band gap, indicating electronic interaction between the dye molecules and the semiconductor NPs. Open-aperture Z-scan results demonstrated strong nonlinear absorption (NLA) behavior dominated by two-photon absorption and excited-state absorption processes. Closed-aperture measurements showed a negative nonlinear refractive (NLR) index, corresponding to self-defocusing behavior. Both the NLA coefficient and the NLR index increased with increasing NP concentration, resulting in a significant enhancement of the third-order nonlinear susceptibility of the nanocomposite systems. In addition, optical limiting measurements revealed a pronounced reduction in the limiting threshold with increasing nanoparticle concentration, demonstrating improved laser attenuation capability. These findings indicate that ZnO@TAIPDI and TiO_2_@TAIPDI nanocomposites are promising candidates for applications in optical limiting, all-optical switching, and advanced photonic devices.

## 1. Introduction

The rapid development of high-power laser systems in scientific, industrial, and military applications has intensified the need for effective optical protection devices [1]. Optical limiters play a crucial role in safeguarding sensitive optical components and human eyes from damage caused by intense laser radiation. These devices operate by exhibiting high optical transparency under low-intensity illumination while significantly reducing transmission when the incident laser intensity exceeds a certain threshold [1,2]. Consequently, the development of materials with strong and controllable linear and nonlinear optical (NLO) responses has become a major focus in modern photonic material research [3].

Organic dyes are prominent optical limiters due to their large nonlinearities and ultrafast optical response [4]. In addition, their molecular structures can be chemically tailored to achieve tunable optical properties for photonic applications [5]. Among these materials, perylenediimide (TAIPDI) derivatives are particularly attractive because of their exceptional photochemical stability, high fluorescence quantum yield, excellent solubility in aqueous and organic media, and strong thermal robustness [6]. While TAIPDI is well-suited for photonic applications like all-optical switching, its intrinsic nonlinear response often falls short of the requirements for high-intensity, ultrafast laser environments [7,8]. Consequently, developing TAIPDI-based hybrid materials has become a primary strategy to enhance these nonlinear optical properties.

One effective approach for improving the linear and nonlinear optical (NLO) characteristics of organic dyes is the incorporation of semiconductor nanoparticles (NPs). Semiconductor NPs exhibit unique size-dependent optical and electronic properties arising from quantum confinement effects [9]. In addition, their high surface-to-volume ratios and strong light–matter interactions make them highly attractive for enhancing optical nonlinearities in hybrid nanocomposite systems [10].

Among various semiconductor nanomaterials, zinc oxide (ZnO) nanoparticles have attracted considerable attention because of their wide band gap, high optical damage threshold, and strong excitonic properties [11]. Titanium dioxide (TiO_2_) nanoparticles are also promising for photonic and nonlinear optical applications owing to their excellent chemical stability and strong interaction with optical radiation [12].

One effective approach to improving the linear and NLO characteristics of organic dyes is to dope them with semiconductor nanoparticles (NPs). The optical and electronic properties of semiconductor NPs are strongly influenced by particle size owing to the presence of quantum confinement effects, in addition to their high surface-to-volume ratios and strong light–matter interactions [9,10]. Among various semiconductor nanomaterials, zinc oxide (ZnO) and titanium dioxide (TiO_2_) NPs have attracted particular interest due to their wide band gaps, high optical damage thresholds, excellent chemical stability, and strong interaction with optical radiation [11,12].

These features make ZnO and TiO_2_ nanostructures highly suitable for integration into hybrid photonic materials designed for several nonlinear optical applications. Pulsed laser ablation (PLA), recognized as a clean, simple, and precisely controllable top-down approach, is employed for the synthesis of ZnO and TiO_2_ NPs. In this technique, a pulsed Nd:YAG laser operating at 532 nm is used to ablate a solid target in a liquid environment, producing stable colloidal NPs. The morphology, size, and optical properties of the generated nanoparticles can be effectively controlled by adjusting several parameters, including laser fluence, pulse duration, wavelength, and the surrounding dielectric medium [13,14]. Compared with conventional chemical synthesis techniques, PLA offers the advantage of producing high-purity NPs without the need for chemical precursors or stabilizing agents, which is particularly beneficial for optical applications where impurities may degrade the nonlinear response.

The incorporation of such semiconductor NPs into organic dye matrices leads to significant modifications in the optical response of the host material. These modifications arise from several mechanisms, including charge transfer processes between the nanoparticles and dye molecules, local electromagnetic field enhancement around the NPs, and exciton–plasmon or exciton–exciton interactions within the hybrid structure [15]. For instance, plasmon–exciton interactions between metallic nanoparticles and dye molecules have been shown to alter optical absorption characteristics and exciton dynamics, leading to enhanced light–matter interactions in hybrid systems [15]. Similarly, charge transfer processes at ZnO–polymer interfaces can modify NLA behavior in nanocomposite films [16]. In addition, theoretical and experimental studies of exciton–plasmon coupling provide a mechanistic explanation for enhanced third-order nonlinear susceptibility χ^(3)^ and modified refractive responses in semiconductor–organic nanostructures [17]. Moreover, the nonlinear optical response of semiconductor–organic nanocomposites may be further optimized through careful control of nanoparticle synthesis and processing conditions, including particle size, crystallinity, and surface properties, which can significantly influence the interfacial optical interactions within the hybrid system [18]. The incorporation of semiconductor NPs may therefore contribute to enhanced third-order nonlinear susceptibility through a combination of intrinsic nanoparticle nonlinearity and possible interfacial interactions with dye molecules.

Despite the considerable progress achieved in hybrid dye–NP systems, several important challenges remain. Relatively limited attention has been devoted to semiconductor NPs doping in TAIPDI derivatives for ultrafast nonlinear optical applications. Furthermore, systematic investigations comparing the influence of different semiconductor NPs on the nonlinear optical response of TAIPDI dye under femtosecond laser excitation remain scarce. In particular, the combined effects of NP type, concentration, and dye–NP interaction mechanisms on third-order nonlinear susceptibility and optical limiting behavior are not yet fully understood. In this context, the present work focuses on modifying the linear and NLO properties of TAIPDI dye through doping with ZnO and TiO_2_ nanoparticles for optical limiter applications. The influence of ZnO and TiO_2_ NPs on both the linear optical characteristics and nonlinear optical response of TAIPDI dye is investigated. The effects of NP type and concentration on the absorption spectra, refractive index, and third-order nonlinear optical parameters of the hybrid system are systematically examined using a UV–visible spectrophotometer and femtosecond Z-scan techniques at an excitation wavelength of 800 nm with an incident power of 1 W. The third-order NLO parameters, including the NLA coefficient (*β*), nonlinear refractive (NLR) index (*n*_2_), and third-order susceptibility (*χ*^(3)^), are experimentally extracted from open- and closed-aperture Z-scan analyses. Particular attention is given to understanding the physical mechanisms responsible for the enhancement of optical limiting performance as a function of ZnO and TiO_2_ NPs concentration, including plasmon-induced local field effects, and energy transfer processes between TAIPDI molecules and semiconductor NPs. The results provide important insights into the design of hybrid PDI-based nanostructures with improved nonlinear optical performance and demonstrate the potential of semiconductor NP doping as an effective strategy for developing advanced optical limiting materials for photonic and optoelectronic applications.

## 2. Materials and Methods

### 2.1. Synthesis of ZnO and TiO_2_ NP Colloids

**Figure 1:**
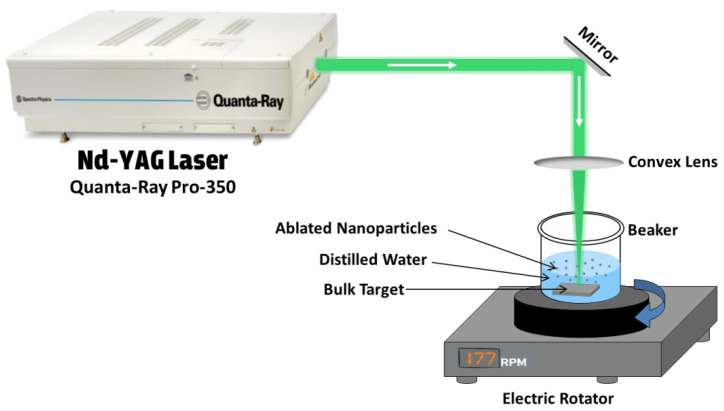
Schematic setup for the synthesis of NPs colloids using a 532 nm Nd:YAG laser ablation system.

The experimental configuration utilized for the synthesis of ZnO and TiO_2_ nanoparticle colloidal suspensions through pulsed laser ablation is depicted in Figure 1. The ablation process was carried out using a second-harmonic Nd:YAG laser system (Spectra Physics–Quanta-Ray PRO 350) emitting at 532 nm (Spectra-Physics, Milpitas, CA, USA). The laser operated with a pulse width of 10 ns and a repetition frequency of 10 Hz, delivering a maximum pulse energy of 1500 mJ while maintaining a near-ideal Gaussian beam distribution (TEM_00_ mode). High-purity zinc and titanium metal targets (>99%) were submerged in 10 mL of distilled water (ablation medium) inside a glass beaker. TiO_2_ NPs were prepared by means of laser irradiation at 532 nm using an average laser power of 250 mW for 15 min, whereas ZnO NPs were synthesized under similar conditions with an average laser power of 200 mW and an irradiation time of 20 min.

Before the ablation process, the surfaces of the zinc and titanium targets were mechanically polished to eliminate oxide films generated by atmospheric exposure. The polished targets were then subjected to ultrasonic cleaning in ethanol, followed by deionized water for 30 min, thereby removing organic impurities and residual surface contaminants. This cleaning method improves the purity of the resulting nanoparticle colloids. The laser beam was then focused onto the target surface using a convex lens with a focal length of 10 cm, producing a focal spot diameter of ~1.75 mm, as estimated using the knife-edge technique. During laser ablation, the beaker was continuously rotated at a speed of 177 rpm by a motorized platform to ensure homogeneous ablation, as well as minimize thermal accumulation and nanoparticle agglomeration. This synthesis technique provides a clean and controllable method for nanoparticle preparation, creating stable ZnO and TiO_2_ colloidal nanoparticles with predominantly spherical morphology while avoiding the use of chemical precursors, reducing agents, or surfactants that may introduce impurities affecting the optical properties of the nanomaterials. It is worth noting that during pulsed laser ablation in distilled water, the freshly generated pure Zn and Ti NPs rapidly react with dissolved oxygen molecules, leading to oxidation and the formation of ZnO and TiO_2_ NPs. This is consistent with the optical behavior, colloidal stability, and TEM morphology of the synthesized ZnO and TiO_2_ NPs that were reported previously for laser ablation in water [19,20].

### 2.2. Preparation of TAIPDI Dye and NPs@TAIPDI Nanocomposites

**Figure 2:**
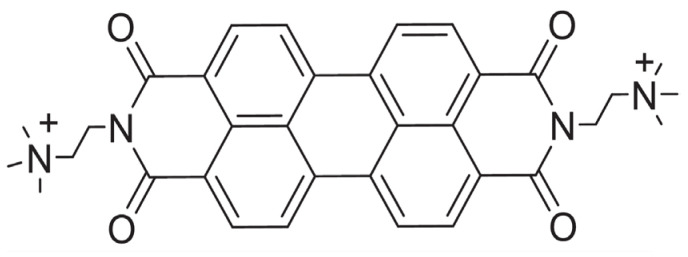
Chemical structure of TAIPDI [8].

The TAIPDI compound was synthesized according to previously reported procedures [17,18]. To obtain a solution with a concentration of 7.2 × 10^−6^ M, the stock dye solution was systematically diluted with distilled water based on the standard dilution equation:

(1) $$C_{1}V_{1}=C_{2}V_{2}$$

where *C*_1_ represents the initial (stock) concentration, *C*_2_ represents the desired final concentration, *V*_1_ is the required stock solution volume, and *V*_2_ is the final volume of the diluted solution. The molecular structure of the synthesized TAIPDI derivative is shown in Figure 2 [8].

To prepare samples with different concentrations, ZnO and TiO_2_ nanoparticles synthesized via PAL were individually diluted using distilled water. For ZnO NPs, concentrations of 4.4, 9.6, 14, and 17.2 mg/L were prepared, while TiO_2_ nanoparticles were diluted to concentrations of 0.85, 2, 2.7, and 6 mg/L. The prepared nanoparticle colloids were combined with the TAIPDI dye solution, resulting in the formation of ZnO@TAIPDI and TiO_2_@TAIPDI hybrid nanocomposite systems. The combinations were subjected to magnetic stirring for 30 min to achieve homogeneous nanoparticle dispersion within the dye solution and to promote effective interaction between the semiconductor nanoparticles and TAIPDI molecules. The colloidal stability of the ZnO@TAIPDI and TiO_2_@TAIPDI nanocomposites is attributed to electrostatic stabilization induced by the ionic trimethylammonium groups of the TAIPDI molecules, which suppress nanoparticle aggregation through repulsive electrostatic interactions [21]. Furthermore, the relatively low nanoparticle concentrations employed in this work reduce the probability of particle agglomeration and precipitation in the aqueous medium [22]. However, to minimize nanoparticle aggregation and maintain homogeneous dispersion in the solution, the samples were continuously mixed using a magnetic stirrer before each scan measurement. This procedure helped to redisperse the nanoparticles uniformly in the aqueous TAIPDI dye solution during the experimental measurements. Therefore, the obtained optical measurements were performed under well-dispersed conditions.

### 2.3. Z-Scan Setup

**Figure 3:**
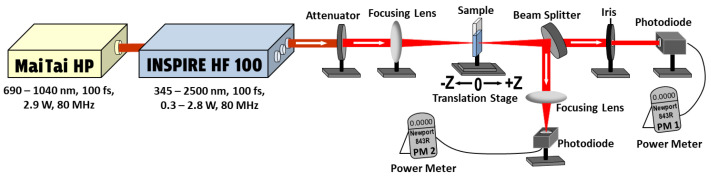
Z-scan experimental setup.

The Z-scan technique was employed to measure the NLO properties of the prepared samples, as schematically illustrated in Figure 3 [23,24]. The measurements were performed using a femtosecond laser system (INSPIRE HF100) pumped by a MAI TAI HP Ti:sapphire laser operating at a wavelength of 800 nm (Spectra-Physics, Milpitas, CA, USA). The laser beam exhibited a Gaussian spatial profile corresponding to the TEM_00_ mode with a beam quality factor M^2^ < 1.1. The laser beam was tightly focused using a convex lens with a focal length of 5 cm in order to achieve high optical intensity at the focal region. Such high intensity is required to induce measurable nonlinear optical effects within the sample. The samples were placed in 1 mm path-length quartz cuvettes mounted on a precision micrometer translation stage, allowing controlled movement of the sample along the propagation direction (z-axis) around the focal region of the laser beam. The transmitted intensity was recorded using a Newport 843R power meter (PM) (Newport, Irvine, CA, USA) as a function of the sample position (z) relative to the focal point (focus). In the closed-aperture (CA) Z-scan setup, an iris with a linear transmittance of *S* = 0.3 was placed before the detector (photodiode) to determine both the sign and magnitude of the nonlinear refractive index *n*_2_, measured via PM1. In contrast, the open-aperture (OA) setup enabled the extraction of the nonlinear absorption coefficient *β* from the variation in transmittance due to intensity-dependent absorption. These measurements were performed using PM2. The experimental transmittance curves obtained from both OA and CA configurations were fitted using conventional theoretical models under the thin-sample approximation framework, enabling accurate extraction of the NLO parameters.

## 3. Results and Discussion

### 3.1. Characterization of ZnO and TiO_2_ NP Colloides

**Figure 4:**
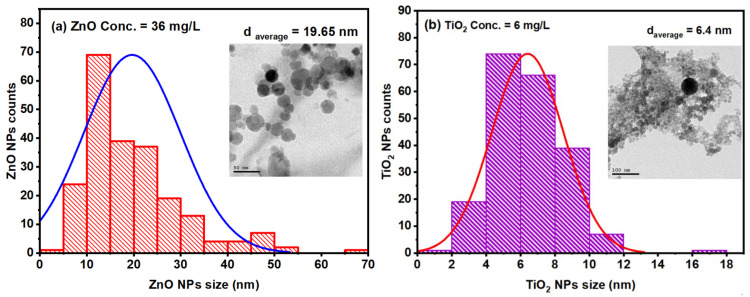
Size distribution histogram for (**a**) ZnO NPs and (**b**) TiO_2_ NPs. The corresponding TEM images of the synthesized ZnO and TiO_2_ spherical NPs are shown as insets. The blue and red solid curves represent Gaussian fitting functions used to estimate the average particle size distribution.

The concentrations of the synthesized ZnO and TiO_2_ NP colloids were determined by means of inductively coupled plasma optical emission spectroscopy (ICP-OES, Agilent 5100 Synchronous Vertical Dual View with a VGA 77 vapor generation accessory, Agilent, Santa Clara, CA, USA). For laser ablation performed at 532 nm with an average power of 250 mW over 15 min, the resulting TiO_2_ colloid concentration was approximately 6 ± 0.6 mg/L. In comparison, ZnO NPs prepared at an average power of 200 mW and over 20 min exhibited a concentration of approximately 36 ± 3.6 mg/L. The morphology and size distribution of the synthesized nanoparticles were characterized by means of high-resolution transmission electron microscopy (HR-TEM, JEM-2100, JEOL, Tokyo, Japan), working at an accelerating voltage of 200 kV. Using ImageJ2 software, particle diameters were extracted from several representative regions of the TEM micrographs. The obtained data were further processed to produce particle size distribution histograms and to determine the average particle diameter. The obtained size distribution histograms, together with the corresponding insets of the TEM images of ZnO NPs and TiO_2_ NPs, are presented in Figure 4a and b, respectively. The analysis confirms that both ZnO and TiO_2_ nanoparticles exhibit predominantly spherical morphology. The average particle size was determined to be 19.65 ± 1.96 nm for ZnO NPs and 6.4 ± 0.64 nm for TiO_2_ NPs. The relatively narrow size distributions indicate that the pulsed laser ablation method provides good control over nanoparticle nucleation and growth processes, resulting in well-dispersed colloidal nanostructures suitable for optical applications.

### 3.2. Linear Optical Properties of ZnO@TAIPDI and TiO_2_@TAIPDI Nanocomposites

#### 3.2.1. UV–Visible Absorption Analysis

**Figure 5:**
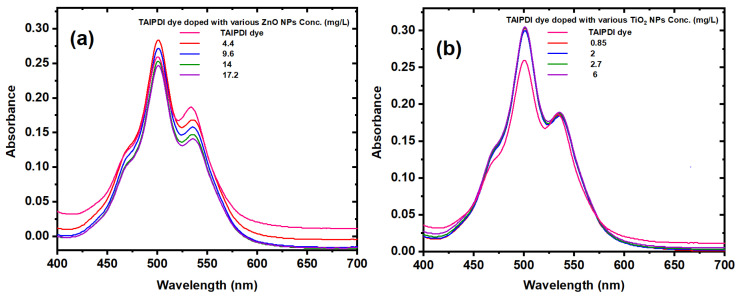
Spectral absorbance of (**a**) TAIPDI dye doped with ZnO NPs (ZnO@TAIPDI nanocomposites) and (**b**) TiO_2_@TAIPDI nanocomposites at different ZnO and TiO_2_ NPs concentrations as a function of wavelength.

A UV–visible spectrophotometer (Model C-7200) (Shimadzu, Kyoto, Japan) was employed to characterize the optical absorption behavior of the ZnO@TAIPDI and TiO_2_@TAIPDI nanocomposites across a broad spectral range extending from 200 to 1100 nm. Figure 4 presents the absorption spectra of (a) ZnO@TAIPDI and (b) TiO_2_@TAIPDI nanocomposites prepared with different nanoparticle concentrations. Figure 5a shows the UV–visible absorption spectra of TAIPDI dye doped with different concentrations of ZnO NPs (4.4, 9.6, 14, and 17.2 mg/L) compared with the pristine TAIPDI dye within the spectral region of 400–700 nm. The spectra reveal that the incorporation of ZnO NPs modifies the absorption intensity of the TAIPDI dye without producing significant shifts in the spectral peak positions. This highlights the controlled tuning of optical properties with nanoparticle concentration. As the ZnO NP concentration increases, a slight decrease in the peak absorbance is observed. This behavior may be attributed to partial electronic coupling between the dye molecules and the ZnO nanoparticles, which can alter the oscillator strength of the electronic transitions and affect the effective absorption cross-section of the dye. Importantly, the overall spectral shape remains nearly unchanged, indicating that the dye molecules retain their molecular integrity without noticeable aggregation or degradation in the presence of ZnO nanoparticles. Figure 5b shows the absorption spectrum of the pristine TAIPDI dye, together with the spectra of the TAIPDI dye doped with TiO_2_ nanoparticles (TiO_2_@TAIPDI nanocomposites) at concentrations of 0.85, 2, 2.7, and 6 mg/L. Similar to the ZnO system, the spectral profiles remain without producing significant shifts in the spectral peak positions, while moderate changes in absorbance intensity are observed with increasing TiO_2_ NPs concentration. The optical absorption spectra of perylenediimide (PDI) dyes are typically characterized by strong and distinct π–π* absorption bands in the visible range due to their extended π-conjugated molecular structure. Upon incorporation of ZnO or TiO_2_ NPs, the local optical environment of the dye molecules is enhanced, leading to variations in the absorption intensity depending on the nanoparticle concentration and dispersion quality. These observations indicate the presence of electronic interactions between the semiconductor NPs and the TAIPDI molecules.

#### 3.2.2. Energy Bandgap and Linear Refractive Index

**Figure 6:**
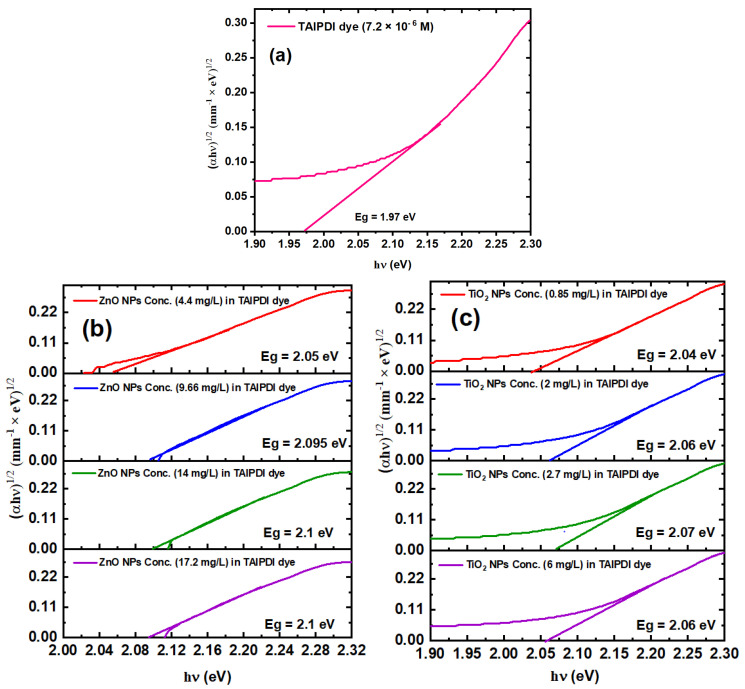
Energy band gaps estimated from Tauc’s plots of (**a**) TAIPDI dye and (**b**) ZnO@TAIPDI and (**c**) TiO_2_@TAIPDI nanocomposites at various ZnO and TiO_2_ NPs colloidal concentrations.

The electronic band structure of the ZnO@TAIPDI and TiO_2_@TAIPDI nanocomposite dye solutions was evaluated by estimating the energy band gap (*Eg*) from the optical absorption spectra through Tauc’s plot equation [25].

(2) $${\left(\alpha h\nu \right)}^{1/2}=a(h\nu -E_{g})$$

where α denotes the linear absorption coefficient, which was determined from the measured absorbance (*A*) and sample thickness (*t*) using the relation α = 2.303 *A*/*t* [26], *hν* represents the photon energy, and *a* is a material-dependent proportionality constant. Figure 6 presents the Tauc’s plots of the ${\left(\alpha h\nu \right)}^{1/2}$ as a function of *hν* for the TAIPDI dye and ZnO@TAIPDI and TiO_2_@TAIPDI nanocomposite solutions [27]. The corresponding *Eg* were determined by linearly extrapolating the absorption-edge region to intercept the photon-energy axis, corresponding to αhν=0. A comparison of the extracted bandgap values presented in Figure 6a for TAIPDI dye with those of the ZnO@TAIPDI and TiO_2_@TAIPDI nanocomposite solutions shown in Figure 6b and Figure 6c, respectively, reveals a gradual increase in the bandgap energy. Specifically, the bandgap increases from 2.05 to 2.10 eV with increasing ZnO NP concentration (4.4–17.2 mg/L), while a more modest increase from 2.04 to 2.06 eV is observed with increasing TiO_2_ NP concentration (0.85–6 mg/L). This slight widening of the optical band gap indicates that the incorporation of semiconductor nanoparticles modifies the electronic structure of the dye–nanoparticle hybrid system, possibly due to weak quantum confinement effects and electronic coupling at the dye–nanoparticle interface. The transmission data were further used to estimate the linear refractive index (*n_o_*) using the Swanepoel relation [28].

(3) $$n_{o}=\frac{1}{T}+{\left(\frac{1}{T^{2}}-1\right)}^{1/2}$$

where *T* is the measured transmittance of the ZnO or TiO_2_@TAIPDI nanocomposite sample.

The refractive index of the ZnO@TAIPDI system decreased from 1.41 to 1.27 as the ZnO NP concentration increased from 4.4 to 17.2 mg/L. In contrast, the refractive index of the TiO_2_@TAIPDI system increased from 1.07 to 1.14 as the TiO_2_ concentration increased from 0.85 to 6 mg/L. These results reveal that the incorporation of semiconductor NPs significantly influences the linear optical response of the dye system, reflecting concentration-dependent modifications of the hybrid medium.

### 3.3. Nonlinear Optical Properties of ZnO@TAIPDI and TiO_2_@TAIPDI Nanocomposites

#### 3.3.1. Open-Aperture Z-Scan Study of Nonlinear Absorption

**Figure 7:**
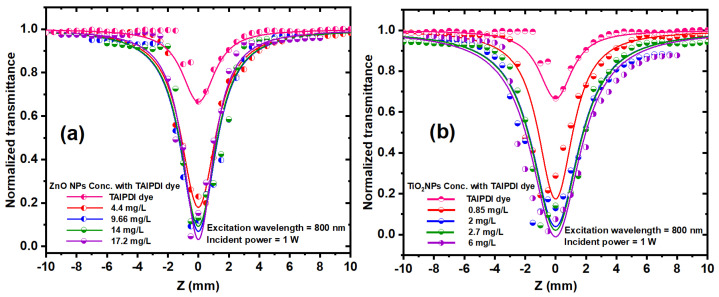
OA Z-scan transmission of TAIPDI dye doped with semiconductor NPs: (**a**) ZnO@TAIPDI and (**b**) TiO_2_@TAIPDI nanocomposites. The dots are the experimental data, whereas the solid curves are the theoretical fits.

The OA Z-scan setup was used to investigate the nonlinear absorption characteristics of TAIPDI dye, ZnO@TAIPDI and TiO_2_@TAIPDI nanocomposites. Figure 7 illustrates the normalized transmittance responses obtained under femtosecond excitation at 800 nm with an incident power of 1 W. Figure 7a and Figure 7b illustrate the effect of ZnO NP concentrations (4.4 to 17.2 mg/L) and TiO_2_ NP concentrations (0.85 to 6 mg/L) on the NLO response of TAIPDI at a fixed dye concentration of 7.2 × 10^−6^ M, respectively. The results show that all samples exhibit symmetric transmission valleys at the focal position (Z = 0), indicating the presence of reverse saturable absorption (RSA) behavior. RSA occurs when the excited-state absorption cross-section exceeds the ground-state absorption cross-section, leading to an increase in absorption with increasing laser intensity. The *Eg* of TAIPDI in water was determined to be 1.97 eV, whereas the photon energy at 800 nm corresponds to approximately 1.55 eV. Therefore, NLA is expected to occur primarily through two-photon absorption (2PA) and excited-state absorption (ESA) processes. It is further observed that the normalized transmittance (*T_OA_*) decreases as the NP concentration increases in the TAIPDI dye.

The nonlinear absorption response at high laser intensities (*I*) is generally expressed using the intensity-dependent absorption relation:

(4) $$\alpha \left(I\right)=\alpha +\beta I$$

where *β* stands for the 2PA coefficient. The experimental OA Z-scan transmittance curves were fitted using the standard theoretical model for NLA under the thin-sample approximation [8,29]:

(5) $$T{\left(z\right)}_{OA}=1-\frac{\beta I_{o}L_{eff}}{2\sqrt{2}\left[1+{\left(Z/Z_{o}\right)}^{2}\right]}$$

where $I_{o}=2P/\pi \omega_{o}^{2}$ represents the peak intensity at the focal point (Z = 0), and $Z_{o}=n_{o}\pi \omega_{o}^{2}/\lambda$ is the Rayleigh length of the focused Gaussian beam. Here, *P* is the peak power, *ω_o_* is the beam waist radius at the focus, and λ is the excitation wavelength. The effective length of the sample, *L_eff_*, is given by $L_{eff}=\left[1-exp(-\alpha L)\right]/\alpha$, where *L* denotes the actual sample thickness. By fitting the experimental OA Z-scan transmittance curves with Equation (5), the NLA coefficient *β* of the TAIPDI-based nanocomposites was extracted, enabling quantitative evaluation of the influence of semiconductor NP concentrations on the NLA response of the hybrid systems.

**Figure 8:**
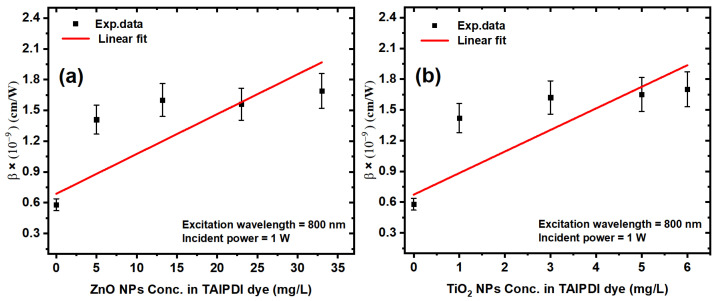
Dependence of *β* on NP concentration for (**a**) ZnO@TAIPDI and (**b**) TiO_2_@TAIPDI nanocomposites. The symbols represent the experimental data, while the solid lines denote the corresponding linear fits.

The extracted *β* values increase systematically with increasing NP concentration, as shown in Figure 8. Several mechanisms are suggested as potential explanations for this improvement. First, the presence of semiconductor NPs introduces additional intermediate energy states, which facilitate ESA and 2PA processes under high-intensity laser excitation. Second, strong electronic interactions and charge-transfer processes at the dye–nanoparticle interface increase the population of excited states, thereby amplifying the NLA response. Furthermore, local field enhancement around the NPs leads to an increased effective optical intensity experienced by the dye molecules. These combined effects may contribute to the enhanced NLA response of the nanocomposite systems as the NP concentration increases. The results confirm that doping organic dyes with semiconductor NPs can effectively modify their NLO performance, highlighting their potential for optical limiting and photonic applications.

#### 3.3.2. Closed-Aperture Z-Scan Study of Nonlinear Refractive Index

The NLR index *n*_2_ of the samples was evaluated using CA Z-scan measurements performed under excitation at 800 nm and 1 W using 100 fs laser pulses with an 80 MHz repetition rate. This configuration enables the determination of both the magnitude andsign of the NLR index through analysis of the characteristic peak–valley features in the CA Z-scan transmittance curves. Notably, the time interval between consecutive pulses (12.5 ns) is much shorter than the time of thermal relaxation of the liquid medium (≥40 µs), given by $t_{relax}=\omega_{o}^{2}/4D$, with *D* being the thermal diffusion coefficient. As a result, the sample is unable to reach thermal equilibrium between successive laser pulses, creating a thermal lens that causes heat to accumulate within the irradiated area. This results in an irregular temperature distribution that changes the refractive-index distribution across the sample. The resulting thermal accumulation affects the CA Z-scan response and can influence the evaluated nonlinear refractive index. Under steady-state thermal equilibrium conditions, the axial change in the refractive index (Δ*n*) is described by the following:

(6) $$\Delta n=\frac{dn}{dT}\frac{I\alpha \omega_{o}^{2}}{4k}$$

where *k* is the thermal conductivity and  dn/dT is the thermo-optic coefficient of the sample.

Typically, electrons are excited from the ground state to higher states upon absorption of one or two photons with identical energy. This excitation process leads to the redistribution of the electronic population among molecular energy levels, which contributes to the refractive index modulation of the medium. Such a population-redistribution process largely governs the refractive nonlinearity of ZnO@TAIPDI and TiO_2_@TAIPDI nanocomposites under femtosecond excitation. The effective number of laser pulses involved in cumulative thermal lensing during the scan is governed by the repetition rate and the spatial position of the sample and is described by the following:

(7) $$\frac{1}{f(Z)}=\frac{a_{fit}LE_{p}F_{l}^{3/2}}{\omega {\left(Z\right)}^{2}}\left[1-\frac{1}{\sqrt{N_{p}}}\right]$$

where *a_fit_* is the fitting parameter given by *a_fit_* = *α* (d*n*/d*T*)/2*k*(π^3^*D*)^1/2^, $\omega \left(z\right)$ is the laser beam radius at the sample position, *F_l_* is the repetition rate, *E_p_* is the pulse energy, and *N_p_* represents the number of incident laser pulses interacting with the sample during the scan. Since *N_p_* = *t* × *F_l_*, where *t* is the exposure time for each TAIPDI sample in this work (*t* ≈ 3 min, *F_l_* = 80 × 10^6^ s^−1^), each sample was irradiated by approximately 1.4 × 10^9^ laser pulses during each scan. Such a large number of pulses significantly enhances cumulative thermal effects, which must be considered in the interpretation of the CA Z-scan data. For *f* ≥ *Z_0_*, the normalized CA transmittance variations (Δ*T*_CA_) depend on the focal length of the induced thermal lens according to

(8) $$\Delta T_{CA}=1+\frac{2Z}{f(Z)}$$

**Figure 9:**
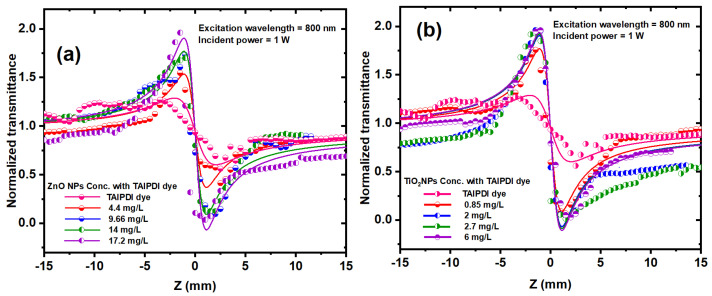
CA Z-scan transmission curves of TAIPDI dye doped with various concentrations of semiconductor NPs: (**a**) ZnO@TAIPDI and (**b**) TiO_2_@TAIPDI nanocomposites. The symbols denote the experimental data, while the solid lines represent the theoretical fits.

Figure 9a and Figure 9b show the CA Z-scan transmittance data of ZnO@TAIPDI and TiO_2_@TAIPDI nanocomposites, respectively, at different NPs concentrations, keeping the dye concentration constant at 7.2 × 10^−6^ M. The figure exhibits a pre-focal peak followed by a post-focal valley, indicating a negative NLR index *n*_2_ (self-defocusing behavior). This behavior is attributed to thermally induced refractive-index gradients resulting from cumulative heating caused by the high repetition rate of the laser pulses that cause the beam to diverge inside the sample. Materials exhibiting negative *n*_2_ are of considerable interest for optical limiting applications [30], all-optical switching [31], NLO devices [32], and soliton generation and propagation [33].

In the OA Z-scan configuration, the aperture was set to allow 30% transmission of the incident light intensity. Such an aperture setting enhances the sensitivity of the CA Z-scan technique to refractive nonlinearities while maintaining sufficient signal-to-noise ratio in the transmitted beam. The thermal-lens parameters were extracted by fitting the experimental data using Equations (7) and (8). At the focal position, the nonlinear phase shift (Δ*φ*) as a function of the thermal focal length is given by the following [34]:

(9) $$\Delta \varphi =\frac{Z_{o}}{2f(0)}$$

where *f*(0) denotes the focal length of the induced thermal lens under the condition where the sample is placed at *Z* = 0. To ensure the validity of the thin-sample approximation (*Z_o_* > *L*), all dye solutions were placed in quartz cuvettes with a 1 mm optical path length. After extracting Δ*φ*, the nonlinear refractive index *n*_2_ can be determined as follows:

(10) $$n_{2}=\frac{\lambda \omega_{o}^{2}\Delta \varphi }{2PL_{eff}}$$

As the concentration of semiconductor NPs increases, a pronounced enhancement in the self-defocusing behavior of the TAIPDI dye doped with ZnO and TiO_2_ NPs is observed. This effect is evidenced by the increasing peak–valley separation in the CA Z-scan traces, indicating a systematic increase in the magnitude of the negative NLR index *n*_2_. The enhanced self-defocusing arises from stronger light–matter interaction induced by the increased NP loading, which modifies the local optical environment of the dye molecules. The increase in NP concentration leads to enhanced electronic polarization and more efficient charge-transfer processes at the dye–NP interface, resulting in a larger refractive nonlinearity. Additionally, thermal contributions associated with increased absorption and nonradiative relaxation become more significant at higher NP concentrations, further contributing to the observed negative *n*_2_ response. The combined effects of electronic and thermal nonlinearities amplify the refractive-index modulation, thereby increasing the peak–valley difference in the Z-scan curves. These results demonstrate that semiconductor NP incorporation provides an effective strategy for controlling the strength of self-defocusing behavior in organic dye systems. Such tunability of the NLR response with NP concentration is highly desirable for designing materials with adjustable nonlinear optical responses for applications in all-optical switching, beam shaping, and photonic devices.

**Figure 10:**
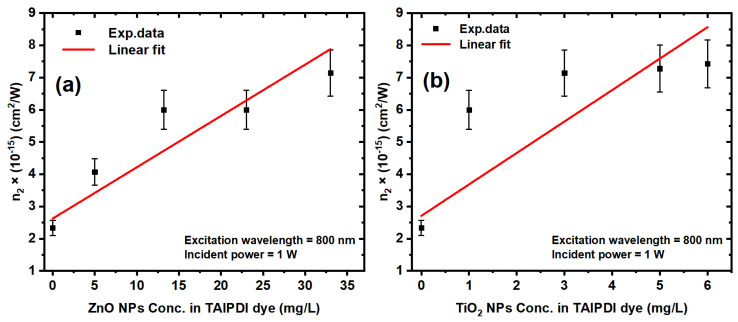
Dependence of the *n*_2_ on semiconductor nanoparticle concentration for (**a**) ZnO@TAIPDI and (**b**) TiO_2_@TAIPDI nanocomposites. The symbols represent the experimental data, while the solid lines correspond to the linear fits.

Figure 10 shows the dependence of the NLR index *n*_2_ on semiconductor NP concentration, as determined using Equations (7)–(10). A systematic increase in the magnitude of *n*_2_ is observed for both ZnO@TAIPDI and TiO_2_@TAIPDI nanocomposite systems with increasing NP concentration. This behavior indicates that higher NP loading strengthens the third-order NLO response of the dye–NP composites. The enhancement of *n_2_* may be associated with increased electronic polarization of the nanocomposite medium and the intrinsic nonlinear refractive response of the semiconductor NPs. Furthermore, the observed behavior may also be influenced by local-field effects and possible interfacial electronic interactions between TAIPDI molecules and the incorporated nanoparticles. The observed enhancement in the nonlinear optical response is consistent with recent studies on engineered Au/TiO_2_ hybrid nanostructures, where strong local electromagnetic field confinement enhances light–matter interactions and nonlinear optical performance [35]. Moreover, interfacial charge-transfer interactions between TAIPDI molecules and the NPs may contribute to a higher population of excited states, resulting in a larger NLR index modulation under intense laser excitation. In addition, increased NP concentration can enhance thermal nonlinear effects due to stronger linear and NLA, which contribute to the observed self-defocusing behavior. The combined electronic and thermal contributions result in a larger negative NLR index, manifested as an increased peak–valley separation in the CA Z-scan curves. These results indicate that the NLR properties of the TAIPDI-based nanocomposites can be effectively controlled by adjusting the NP concentration, providing a practical strategy for tailoring the optical response of hybrid systems. Consequently, these nanocomposites exhibit promising potential for applications in optical limiting, beam shaping and control, all-optical switching, and advanced photonic devices requiring tunable third-order nonlinear optical properties. It was worth noting that the enhancement of β is mainly associated with increased excited-state population generated through interfacial charge-transfer interactions between TAIPDI molecules and semiconductor nanoparticles. These interactions introduce additional electronic states that facilitate sequential photon absorption and ESA processes under femtosecond excitation. In addition, the observed negative NLR index originates from combined electronic and thermal nonlinear contributions.

At higher nanoparticle concentrations, the enhancement of *β* and *n*_2_ tends to approach saturation behavior. This may result from partial saturation of interfacial charge-transfer interactions and local field effects at elevated nanoparticle loading, in addition to increased scattering and screening effects that limit further nonlinear enhancement. The plotted linear fits in Figure 8 and Figure 10 were intended as guides to illustrate the general concentration dependence.

#### 3.3.3. Third-Order Nonlinear Susceptibility (χ^(3)^)

The real and imaginary parts of the third-order nonlinear susceptibility $\chi^{\left(3\right)}$ were calculated from the experimentally obtained values of *n*_2_ and *β* values, respectively, using the following relations [36]:

(11) $$Re\left[\chi^{\left(3\right)}\right]=\frac{n_{o}}{3\pi }n_{2},Im\left[\chi^{\left(3\right)}\right](esu)=\frac{{10^{-7}c\lambda n}_{o}}{96\pi^{2}}\beta$$

where *c* is the speed of light. The total magnitude of the susceptibility is given by the following [29]:

(12) $$\left|\chi^{\left(3\right)}\right|=\sqrt{\left({Re\left[\;\chi^{\left(3\right)}\right]}^{2}+{Im\left[\chi^{\left(3\right)}\right]}^{2}\right)}$$

The absolute values of $\chi^{\left(3\right)}$ are determined at different concentrations of ZnO and TiO_2_ NPs and summarized in Table 1. A pronounced enhancement of $\chi^{\left(3\right)}$ was observed with increasing nanoparticle concentration, particularly for TiO_2_@TAIPDI nanocomposites. The maximum susceptibility was obtained for the sample containing 6 mg/L TiO_2_ nanoparticles, which exhibits nearly a three-fold increase compared with the pristine TAIPDI dye solution. This enhancement may originate from the combined contribution of the intrinsic nonlinear optical response of the semiconductor NPs together with possible local-field enhancement and interfacial electronic interactions within the nanocomposite system. Such local field enhancement increases the effective interaction between the incident laser field and the π-conjugated electronic system of the TAIPDI molecules, thereby strengthening NLA and refraction processes. Additionally, interfacial charge transfer and exciton–exciton interactions may further contribute to the observed improvement in the NLO response of the nanocomposite system.

### 3.4. Optical Limiting Performance

**Figure 11:**
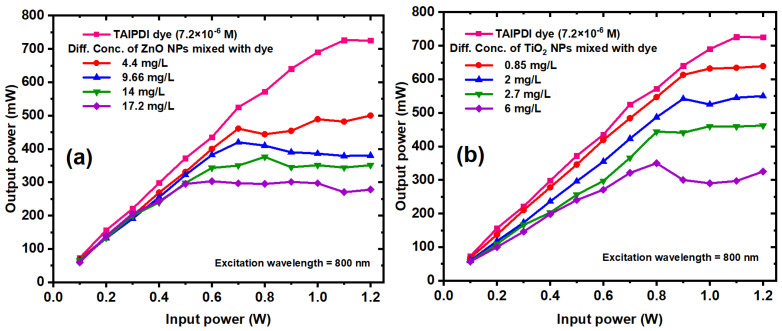
Optical limiting behavior of TAIPDI doped with various concentrations of semiconductor NPs: (**a**) ZnO@TAIPDI and (**b**) TiO_2_@TAIPDI nanocomposites. The samples were positioned at the laser focus, and the aperture was fully open during measurements.

The optical limiting (OL) behavior of TAIPDI, ZnO@TAIPDI, and TiO_2_@TAIPDI nanocomposites was evaluated using the same fs laser setup applied for the Z-scan measurements. The experimental measurements were carried out by placing the sample at the focal position of the lens. At a wavelength of 800 nm, which is beyond the primary absorption region of TAIPDI (470–540 nm), the transmitted power was recorded while varying the incident laser power. Figure 11a and Figure 11b present OL curves for different concentrations of ZnO NPs (4.4–17.2 mg/L) and TiO_2_ NPs (0.85–6 mg/L), respectively. A linear relationship between input and output power is observed at low incident powers, confirming a linear transmission regime. As the input power increases beyond a threshold value, the transmission becomes saturated, indicating the onset of leading to OL behavior. As the nanoparticle concentration increases, the optical limiting response becomes stronger. This enhancement is accompanied by a decrease in the OL threshold from ~700 mW at low NP concentration to ~300 mW at high NP concentration, indicating improved laser protection efficiency. The improved optical limiting behavior is mainly attributed to strong nonlinear absorption processes, including RSA and 2PA, as evidenced by OA Z-scan measurements (Figure 7). At higher laser intensities, ESA becomes the dominant process, leading to effective clamping of the transmitted power. The improved NLA observed in ZnO@TAIPDI and TiO_2_@TAIPDI composites may be associated with the intrinsic nonlinear absorption of the NPs together with possible local electromagnetic-field enhancement induced by surface plasmon resonance (SPR). Furthermore, the incorporation of semiconductor NPs enhances light–matter interactions within the composite medium through local electromagnetic field modulation and increased scattering, which effectively extends the optical path length and raises the probability of NLA events. In addition, localized plasmonic modes generated by the NPs intensify the interaction within the matrix and increase the excitation probability of TAIPDI molecules. Efficient energy transfer between the NPs and the dye molecules further strengthens the NLO response, demonstrating the key role of plasmonic effects in enhancing the optical performance of the nanocomposites. Accordingly, the observed improvement in optical limiting efficiency with increasing NP concentration results from the combined contributions of nonlinear absorption, plasmonic field enhancement, and light scattering from semiconductor nanoparticles [37,38]. These results demonstrate that ZnO@TAIPDI and TiO_2_@TAIPDI nanocomposites exhibit strong, tunable and concentration-dependent optical limiting performance, making them promising candidates for laser protection devices, optical switches, and nonlinear photonic systems.

## 4. Conclusions

This work focuses on the investigation of the linear, nonlinear, and optical limiting characteristics of the perylenediimide derivative TAIPDI dye doped with semiconductor NPs (ZnO and TiO_2_), which were systematically investigated under 800 nm femtosecond laser excitation. ZnO and TiO_2_ NPs were successfully synthesized using the PLA technique, yielding stable spherical colloidal NPs suitable for incorporation into the TAIPDI dye. The incorporation of ZnO and TiO_2_ NPs into the TAIPDI dye significantly modified both the linear and nonlinear optical responses. UV–visible absorption measurements revealed concentration-dependent variations in the optical absorption characteristics. Z-scan measurements demonstrated that the ZnO@TAIPDI and TiO_2_@TAIPDI nanocomposites exhibit pronounced RSA behavior together with a negative NLR index, confirming the presence of strong third-order nonlinear optical effects. Both the NLA coefficient *β* and the NLR index *n*_2_ increased with increasing semiconductor NPs concentration, leading to a considerable enhancement of the overall third-order nonlinear susceptibility. The enhancement of the nonlinear optical response observed with increasing nanoparticle concentration reflects the important role of semiconductor NPs in modifying the optical behavior of the TAIPDI-based nanocomposites. Furthermore, the optical limiting measurements demonstrated a significant reduction in the limiting threshold with increasing ZnO and TiO_2_ NP concentration, indicating improved optical limiting efficiency. Overall, the results demonstrate that semiconductor NP incorporation with TAIPDI provides an effective and versatile strategy for tailoring the nonlinear optical properties of perylenediimide-based materials. The tunable third-order nonlinear response and improved optical limiting behavior observed in the ZnO@TAIPDI and TiO_2_@TAIPDI nanocomposites highlight their strong potential for applications in optical limiting devices, all-optical switching, and advanced photonic technologies.

## Figures and Tables

**Table 1:** Summary of the NLO properties of ZnO@TAIPDI and TiO_2_@TAIPDI nanocomposites at different NP concentrations.

Samples	NPs Conc. (mg/L)	*ꞵ* × 10^−9^ (cm/W)	*n*_2_ × 10^−15^ (cm^2^/W)	Re [$\chi^{\left(3\right)}]$ × 10^−16^ (esu)	$Im\left[\chi^{\left(3\right)}\right]$ × 10^−13^ (esu)	$\chi^{\left(3\right)}$ × 10^−13^ (esu)
ZnO@TAIPDI nanocomposites	0	0.58	2.34	3.05	2.22	2.22
	4.4	1.41	4.07	5.83	6.21	6.21
	9.66	1.6	6	7.62	5.8	5.8
	14	1.56	6	7.25	5.2	5.2
	17.2	1.69	7.14	8.63	5.55	5.55
TiO_2_@TAIPDI nanocomposites	0.85	1.42	6	8.91	7.04	7.04
	2	1.62	7.14	10.68	8.14	8.14
	2.7	1.65	7.28	11.03	8.51	8.51
	6	1.7	7.43	11.4	9	9

## Data Availability

The original contributions presented in this study are included in the article. Further inquiries can be directed to the corresponding author.
